# Supplementary material for: In-situ cross-linking strategy for efficient and operationally stable methylammoniun lead iodide solar cells
Source: Nat Commun. 2018 Sep 18;9:3806. doi: 10.1038/s41467-018-06204-2 (PMC6143610; doi:10.1038/s41467-018-06204-2)
Supplement: Supplementary file 1 — Supplementary Information [file 41467_2018_6204_MOESM1_ESM.pdf]

SUPPLEMENTARY INFORMATION

In-Situ Cross-linking Strategy for Efficient and Operationally Stable Methylammoniun Lead Iodide Solar Cells

Xiaodong Li[1], Wenxiao Zhang[1,2], Ying-Chiao Wang[1], Wenjun Zhang[1,2], Hai-Qiao Wang[1,2] and Junfeng Fang[1,2]*

[1] Ningbo Institute of Materials Technology and Engineering, Chinese Academy of Sciences, Ningbo, 315201, China;

[2] University of Chinese Academy of Sciences, Beijing 100049, China

*E-mail: fangjf@nimte.ac.cn

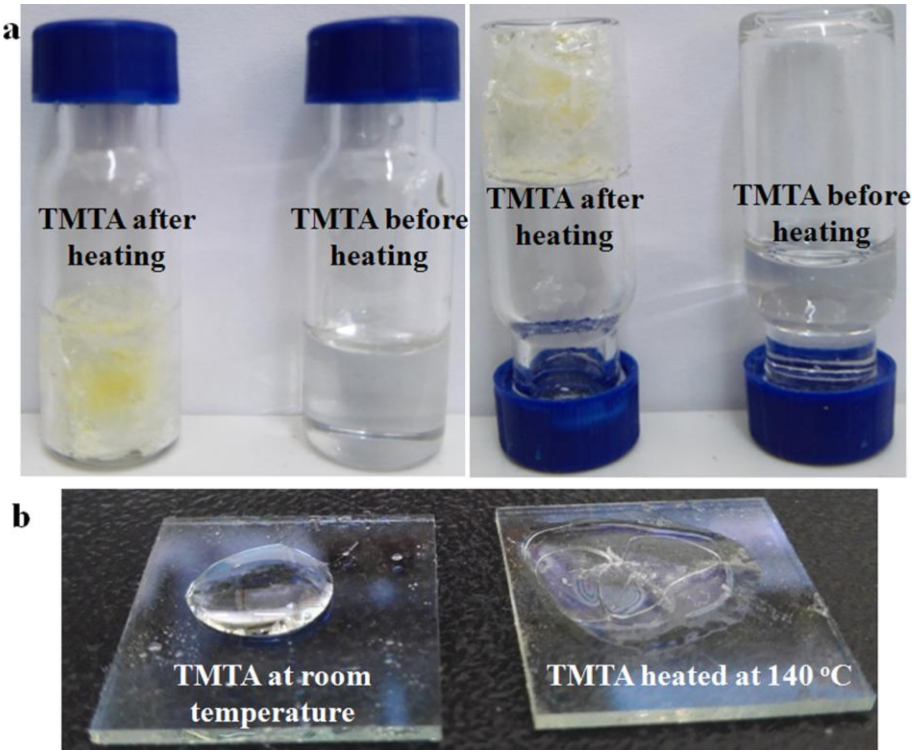

Supplementary Figure 1 │Photographs of TMTA. (a) Photographs of TMTA in reagent bottle before and after heating. TMTA is a liquid at room temperature and becomes a solid after thermal heating. (b) Photographs of TMTA on glass/ITO substrate. After heated at 140 ^{o}C, the liquid TMTA become a robust solid, indicating the cross-linking of TMTA.

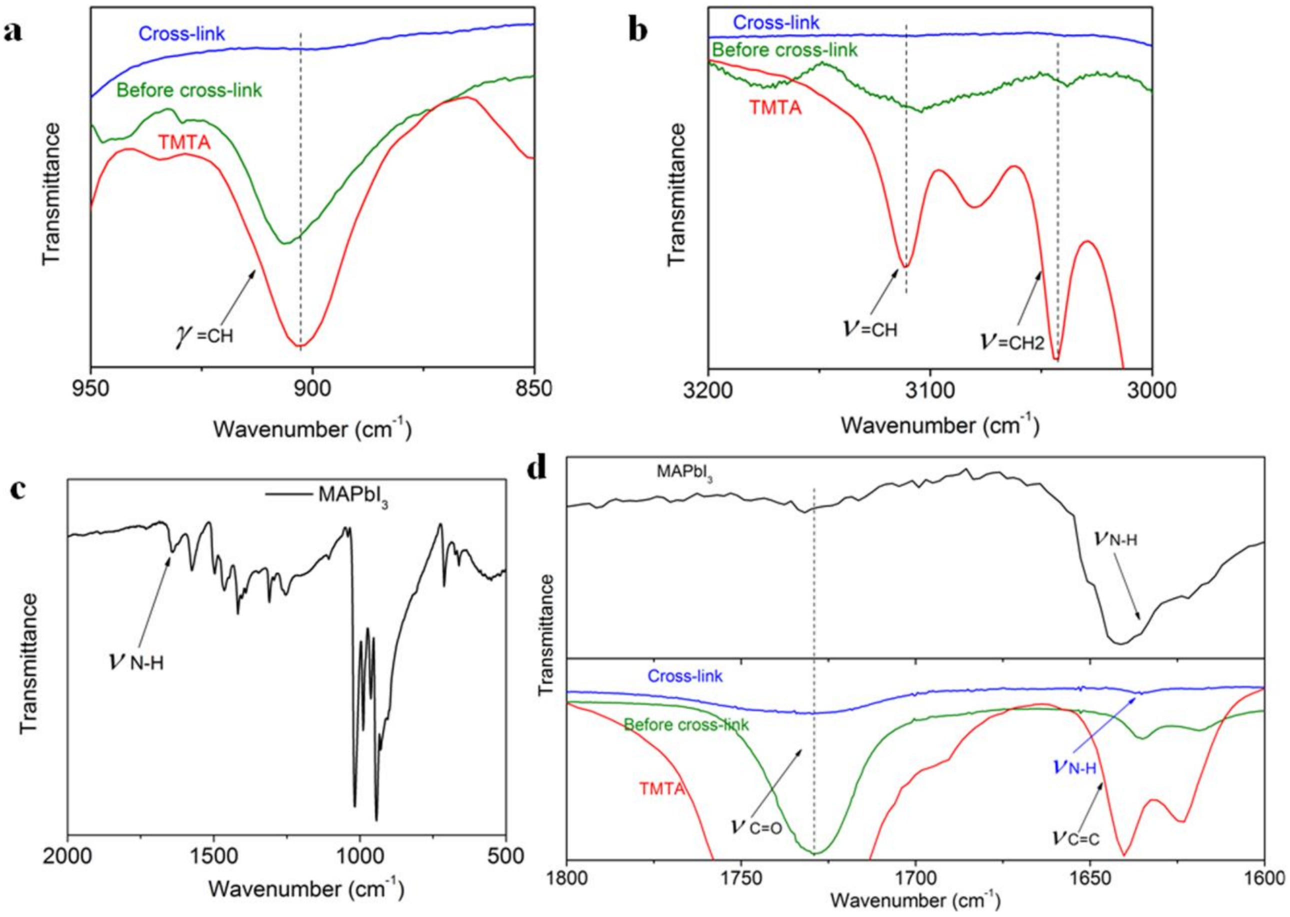

Supplementary Figure 2 | Magnified FTIR spectra. (a) The C-H bending vibration of $\gamma_{=CH2}$; (b) The C-H stretching vibration of $\nu_{=CH}$ and $\nu_{=CH2}$; (c) FTIR spectra of $MAPbI_3$ powder, the arrow indicates the vibration ascribed to N-H groups; (d) The C-C stretching vibration of $\nu_{C=C}$.

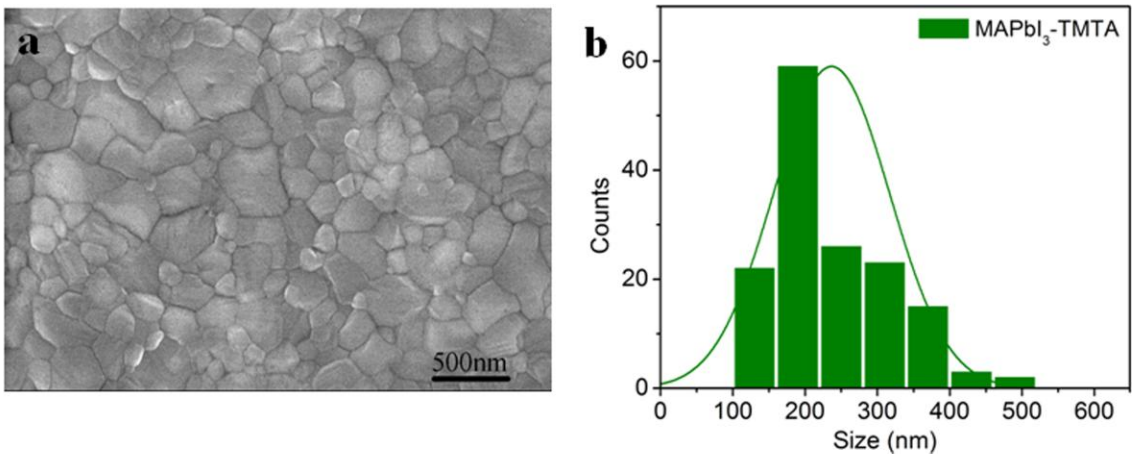

Supplementary Figure 3│Morphological analysis of $MAPbI_3$-TMTA films. (a) Top-view SEM image of $MAPbI_3$-TMTA with magnification of 35k. (b) Grain size distributions estimated from the SEM image in (a) using Nano measurer 1.2 software.

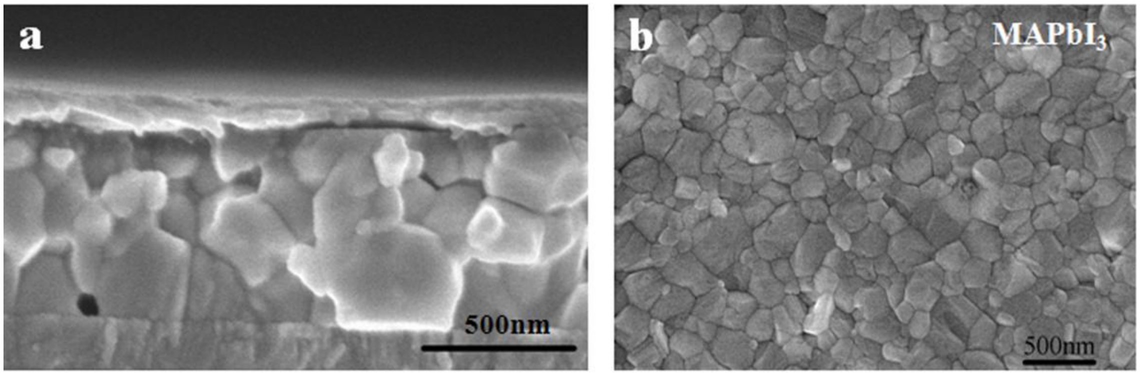

Supplementary Figure 4│Morphology characterisation of control $MAPbI_3$ films. (a) Cross-section SEM image with magnification of 60k and (b) Top-view SEM image with magnification of 35k.

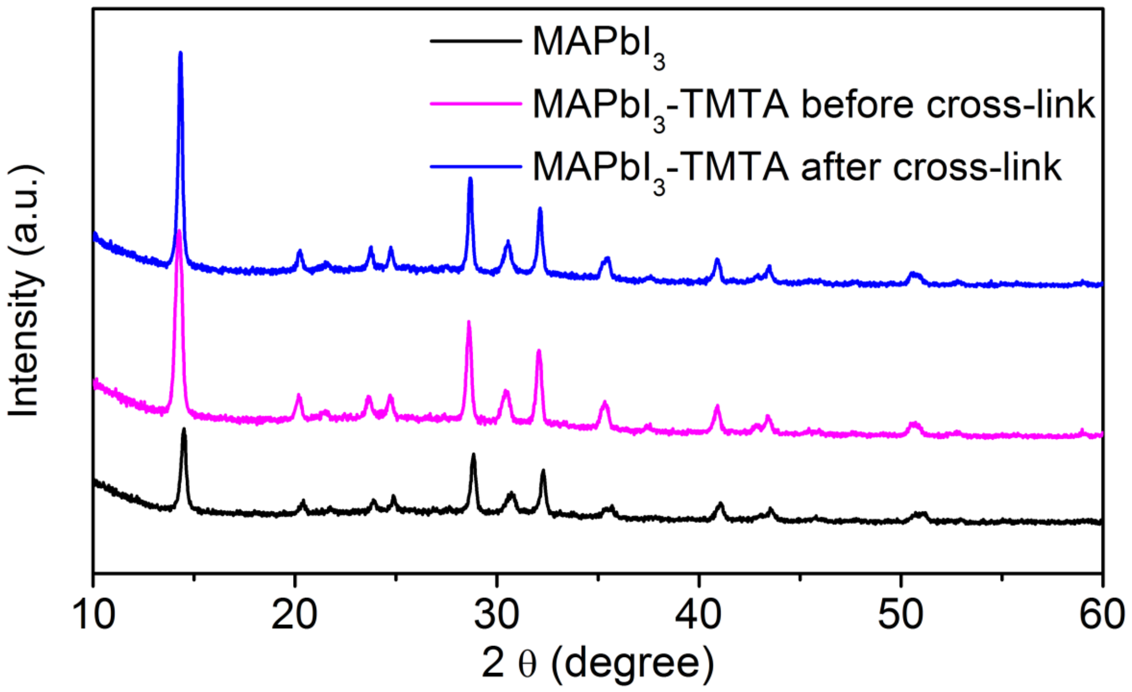

Supplementary Figure 5│X-ray diffraction (XRD) patterns of perovskite films. $MAPbI_3$-TMTA exhibits the similar XRD patterns to $MAPbI_3$ film and no shift in diffraction angle is observed, indicating that the additive of TMTA will not embed into the crystal lattice of perovskite. In addition, no obvious difference is observed in the XRD patterns of $MAPbI_3$-TMTA films before and after cross-linking.

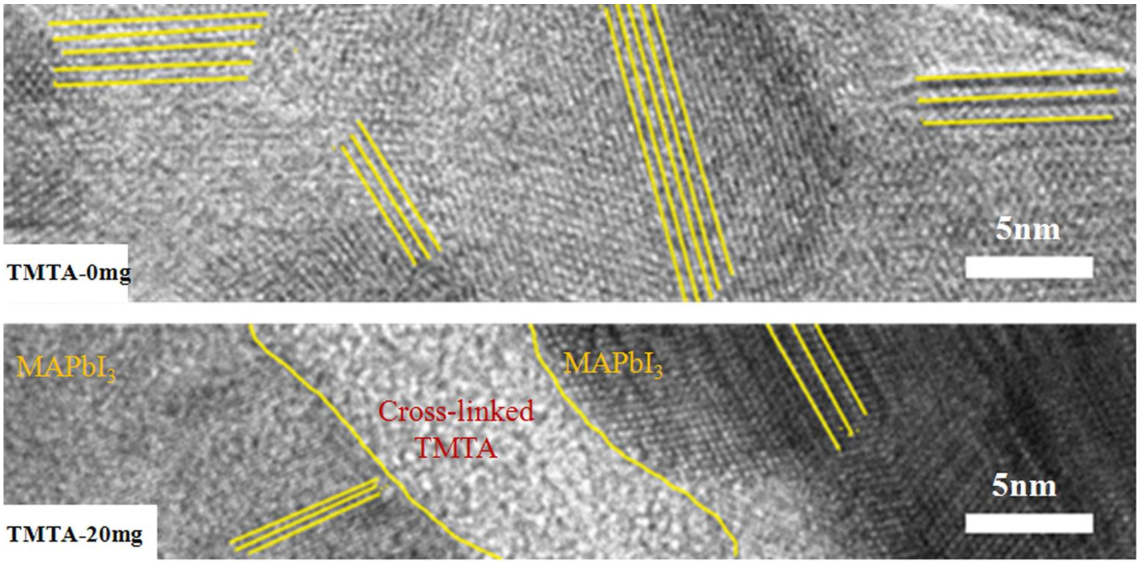

Supplementary Figure 6│Detailed views of HRTEM. In $MAPbI_3$-TMTA, amorphous TMTA is clearly observed among crystalline $MAPbI_3$ grains.

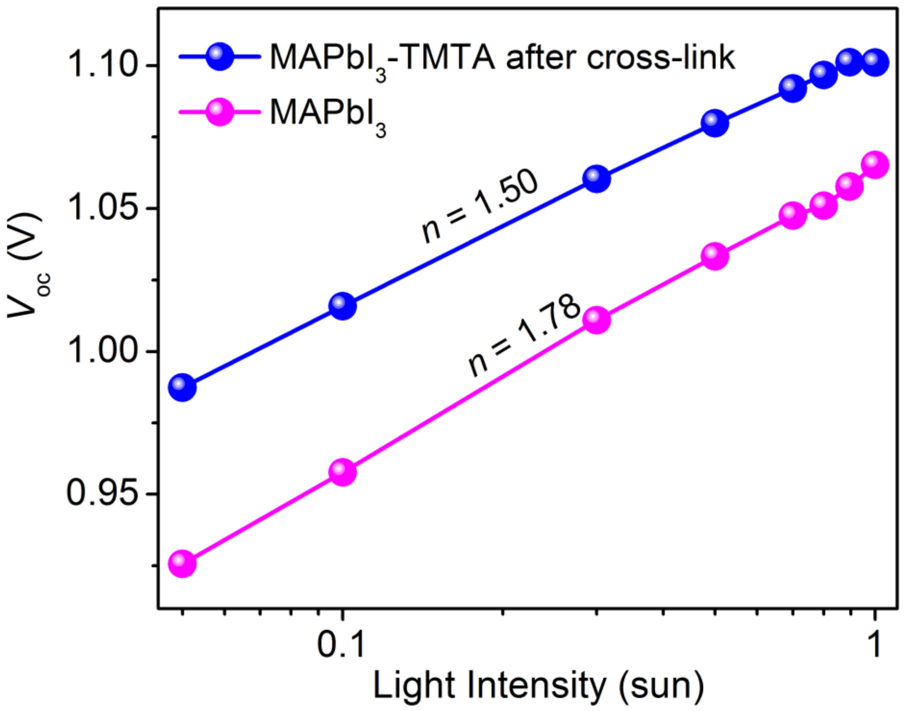

Supplementary Figure 7 | The fitting of devices V_{oc} with light intensity. The intensity dependence of devices V_{oc} is fitted using the following equation:

$n = \frac{q}{2.303\kappa T}\frac{dV_{oc}}{d\lg\rho}$, where n is ideality factor, q is the magnitude of electron charge, κ is Boltzmann constant, T is absolute temperature, ρ is light intensity. The lower value of ideality factor (n) indicates the smaller recombination in $MAPbI_3$-TMTA after cross-link.

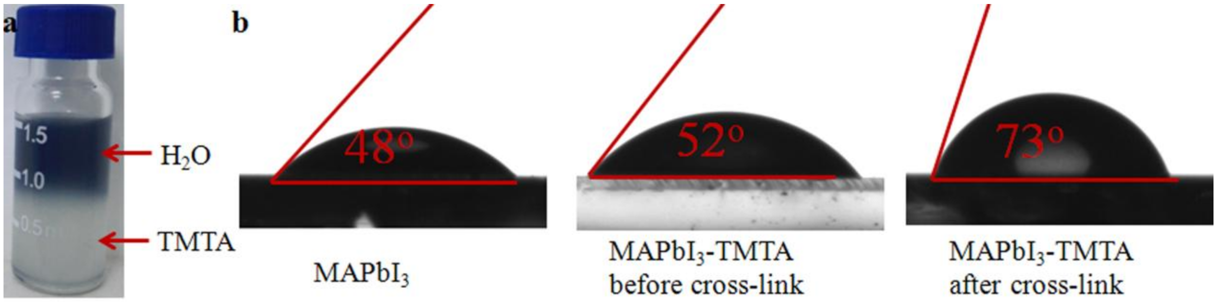

Supplementary Figure 8 | Hydrophobic property of TMTA and contact angle measurement. (a) Hydrophobic property of TMTA. TMTA is mixed with H_2O (some PEDOT:PSS is added to H_2O to increase contrast) with stirring and then put in air overnight. The mixture becomes two layers spontaneously, indicating the hydrophobic nature of TMTA. (b) The contact angle of H_2O on $MAPbI_3$, $MAPbI_3$-TMTA before and after cross-link. After heated at 140 ^{o}C, the $MAPbI_3$-TMTA film exhibits significantly increased contact angle with H_2O, indicating the cross-linking polymerization of TMTA from another point of view. In addition, the increased contact angle of H_2O will block the moisture penetration into bulk perovskite film, thus improving the devices stability in air.

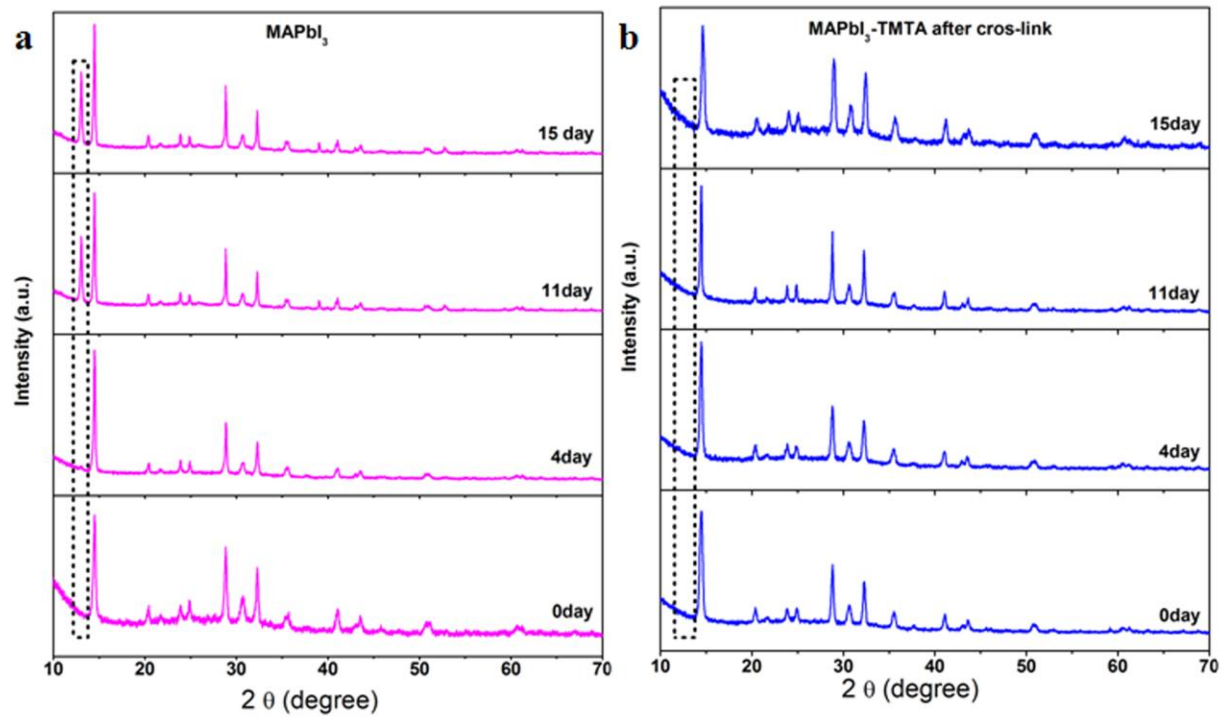

Supplementary Figure 9│XRD evolution of perovskite films exposed to air. (a) XRD patterns of control $MAPbI_3$ films and (b) XRD patterns of $MAPbI_3$-TMTA after cross-link when storage in air (relative humidity: 45-60%). The marked peak in dash rectangle is the characteristic pattern of PbI_2. For control $MAPbI_3$ films, strong PbI_2 pattern appears after storage for 11 days, indicating the decomposition of $MAPbI_3$. However, in $MAPbI_3$-TMTA films after cross-link, no PbI_2 pattern is observed even after 15 days, demonstrating their excellent water-resistive properties, which is beneficial to the devices stability in air conditions.

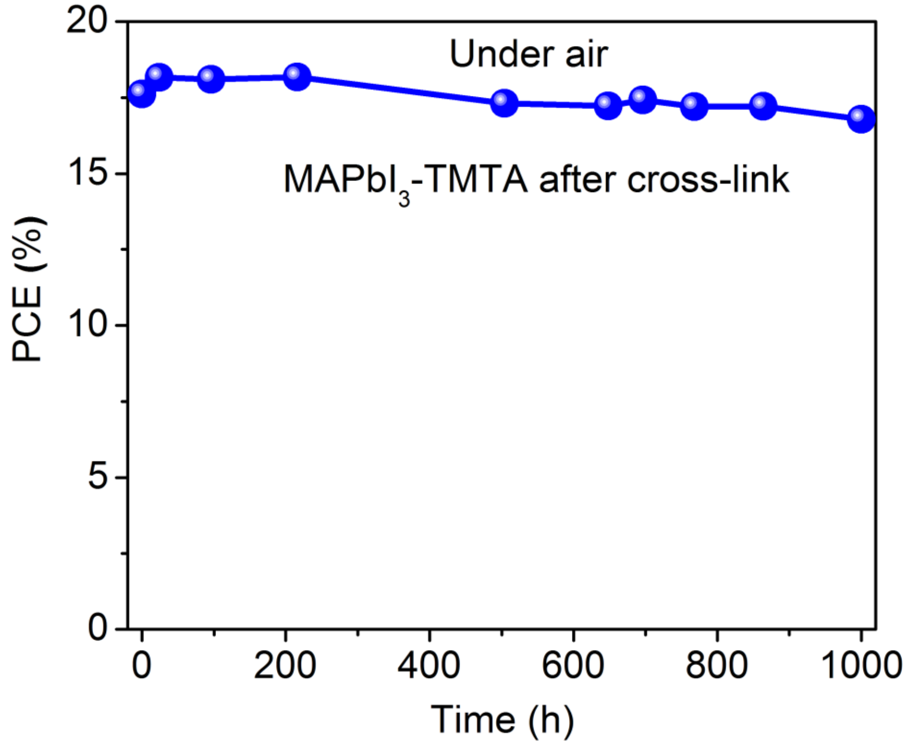

Supplementary Figure 10 | Air stability of PSCs with $MAPbI_3$-TMTA after cross-link. The initial devices efficiency is 17.6% and can be increased to 18.2% after storage for 24 hours. The data shown in Fig. 4a is normalized at the highest efficiency value (18.2%). After storage for 1000 hours, the devices efficiency is slightly decreased to 16.8%, retaining 92% of the highest efficiency value.

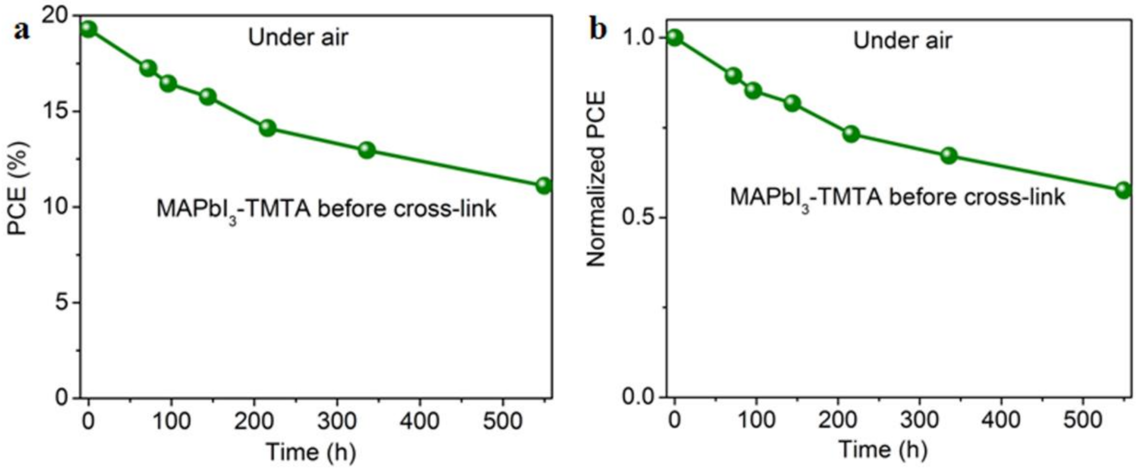

Supplementary Figure 11 | Air stability of PSCs with $MAPbI_3$-TMTA before cross-link. (a) Device efficiency and (b) normalized efficiency evolution when storage in air. The initial device efficiency is 19.3%. It degrades rapidly and the efficiency is 11.1% after 550 hours.

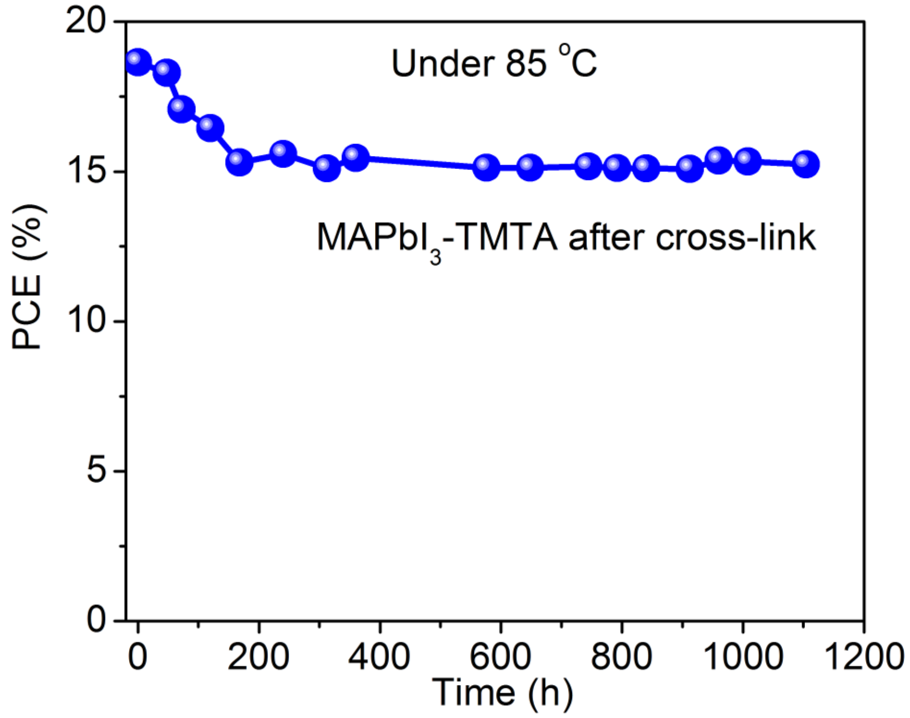

Supplementary Figure 12 | Thermal stability of PSCs with $MAPbI_3$-TMTA after cross-link. The device is stored in glovebox and put on a hotplate setting at 85 °C. The initial efficiency is 18.6%, and it rapidly degrades to 15.3% in first 170 hours. However, in the next 930 hours, almost no degradation is observed.

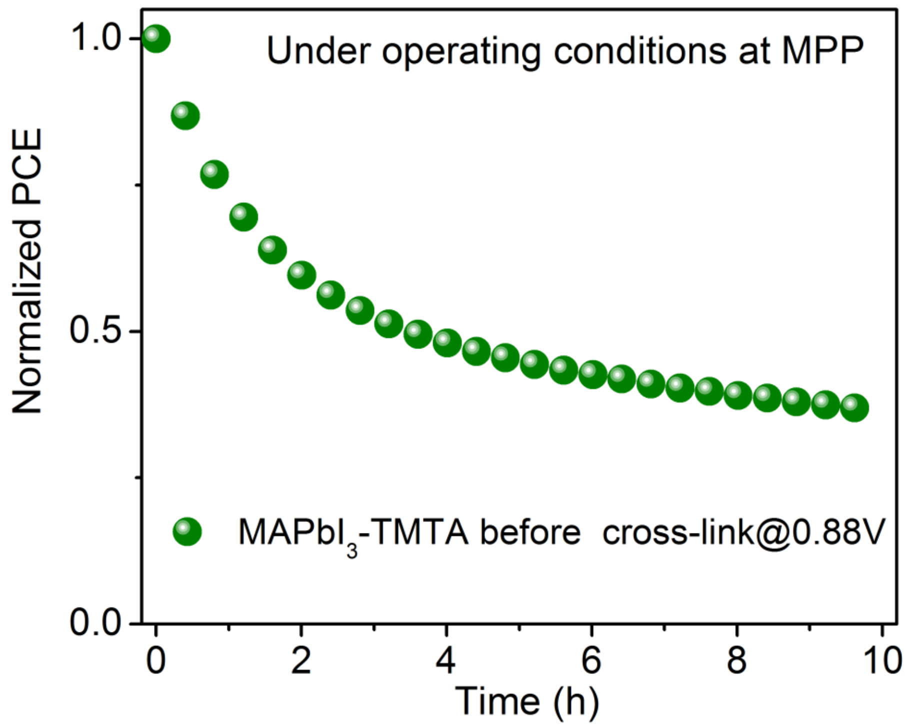

Supplementary Figure 13 | Operational stability of PSCs with $MAPbI_3$-TMTA before cross-link. The device is examined at maximum power point (constant bias of 0.88 V) under AM 1.5G illumination in glovebox.

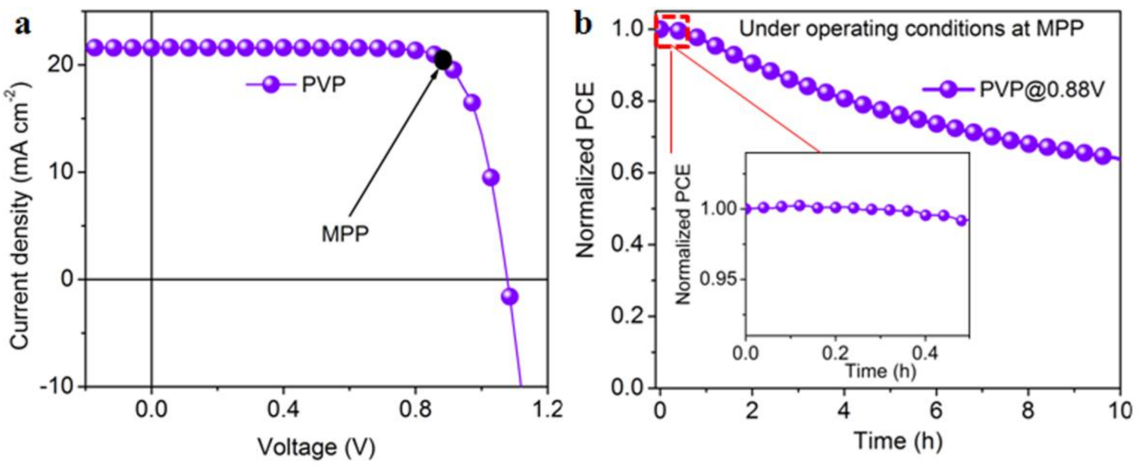

Supplementary Figure 14 | Device performance and operational stability of PSCs with liner polymer PVP. (a) *J-V* curve of PSCs with PVP. The device efficiency is 18.1% with V_{oc} of 1.08 V, J_{sc} of 21.60 mA/cm^2 and FF of 77.9%. The voltage at MPP is 0.88 V as marked in the *J-V* curve. (b) Operational stability of PSCs with PVP at MPP (constant load of 0.88 V) under continuous AM 1.5G illumination in glovebox. Inset: the enlarged operational stability at first 0.5 hour (1800 seconds). At first 0.5 hour, almost no degradation is observed as 99% of the initial efficiency is retained. However, with time expended, the degradation starts to accelerate and after 10 hours, only 60% of the initial efficiency is retained.

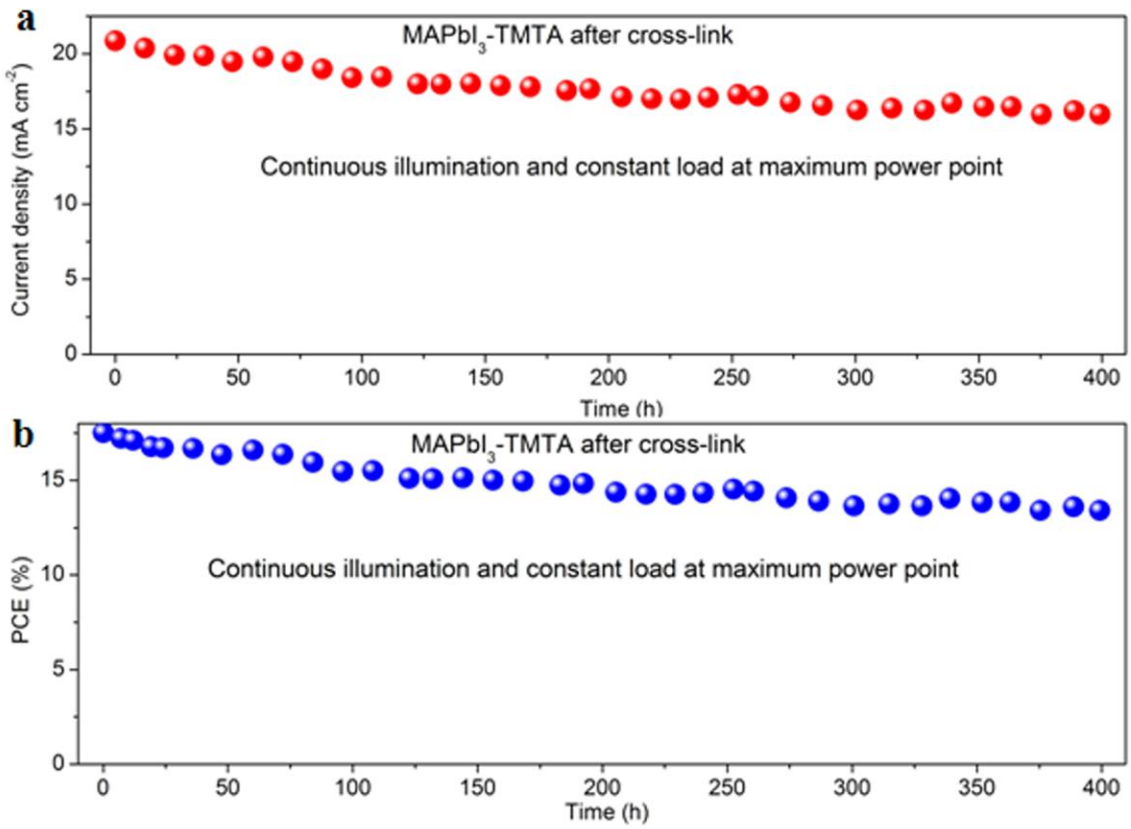

Supplementary Figure 15 | Operational stability of PSCs based on $MAPbI_3$-TMTA after cross-link. (a) Current density variation during the MPP tracking. (b) Non-normalized PCE variation during the MPP tracking. For MPP tracking, the PSCs are applied on a bias of 0.84V (acting as load at MPP) under continuous AM 1.5G illumination. The current density during the MPP tracking is recorded and the PCE is calculated through multiplying the current density by the applied bias of 0.84 V.

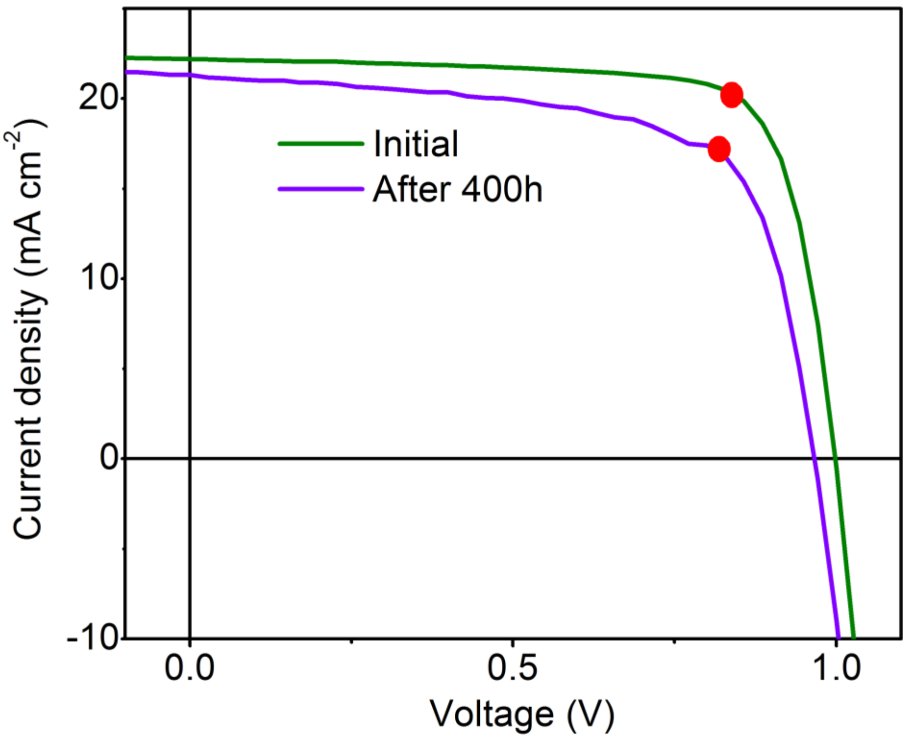

Supplementary Figure 16 | *J-V* curve variation in cross-linked $MAPbI_3$-TMTA devices before and after MPP tracking. The initial PSCs show an efficiency of 17.1% with the MPP voltage of 0.84 V as marked in *J-V* curve. After MPP tracking for 400 hours, the device efficiency decreases to 13.9%. Note that the MPP voltage also decreases to 0.82 V after 400 hours. However, the load of 0.84 V is continuously applied on the PSCs. Just due to this MPP voltage variation, there is a distinction between MPP tracking and *J-V* curve as shown in Fig. 4c.

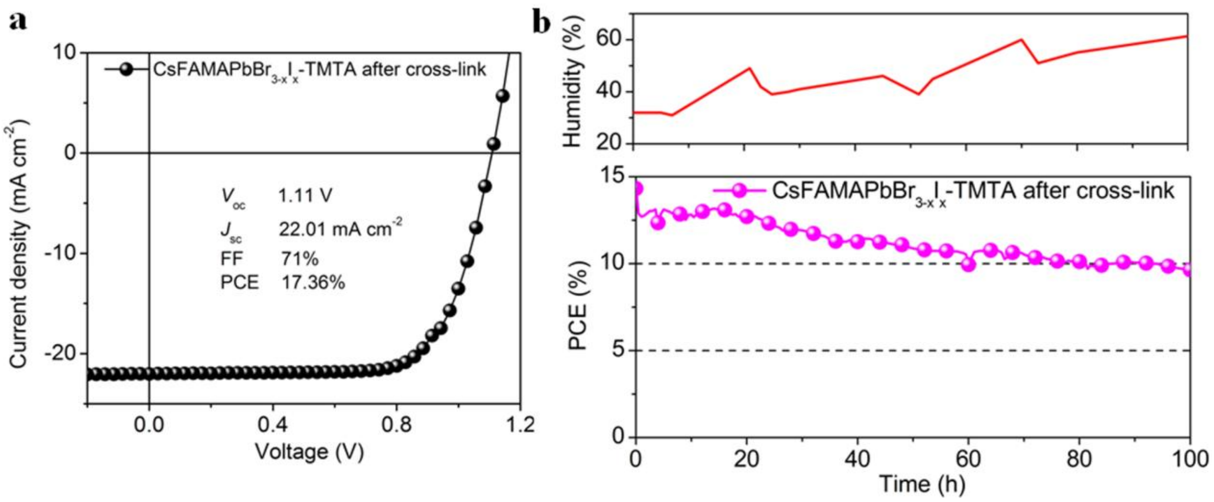

Supplementary Figure 17│*J-V* curve and operational stability of $CsFAMAPbBr_{3-x}I_3$ with TMTA after cross-link. (a) *J-V* curve of $CsFAMAPbBr_{3-x}I_3$ device with TMTA after cross-link; (b) Operational stability of $CsFAMAPbBr_{3-x}I_3$ device in humidity air (relative humidity: 30-60%) without encapsulation. Different to $MAPbI_3$, the operational stability of $CsFAMAPbBr_{3-x}I_3$ device is conducted according to the recommended method by Michael Grätzel and Wolfgang Tress[1]. The device is linked with an electronic load to make sure that the device is always working at MPP and the *J-V* curves are recorded every 30 minutes. The operational stability of $CsFAMAPbBr_{3-x}I_3$ device is conducted under LED lamp.

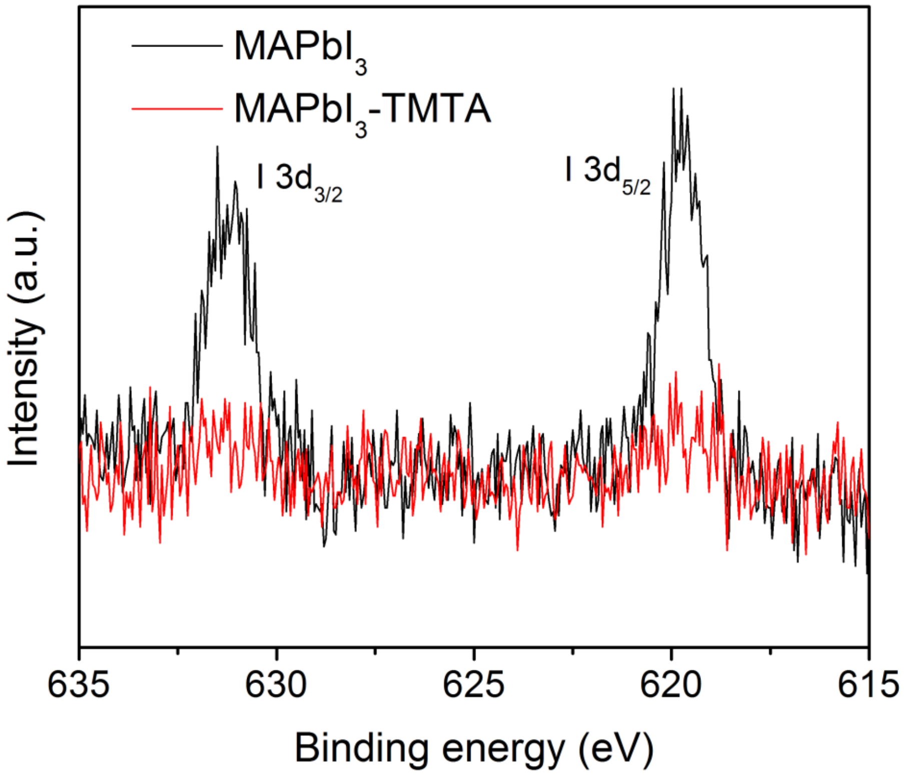

Supplementary Figure 18 | X-ray photoelectron spectroscopy (XPS). The PSCs operates at MPP under illumination for 60 hours. The Cu electrode is removed from the devices with tape to expose the surface of between BCP and Cu. Then XPS measurement is conducted at this surface. In $MAPbI_3$ devices, the characteristic peaks of I $3d_{5/2}$ and I $3d_{3/2}$ appears at 619 and 631 eV respectively. While in $MAPbI_3$-TMTA device, almost no I signal appears.

**Supplementary Table 1│The device parameters of PSCs with control $MAPbI_3$, $MAPbI_3$-TMTA before and after cross-link.**				
**Perovskite**	**V_{oc} (V)**	**J_{sc} (mA/cm^2)**	**FF (%)**	**PCE (%)**
Control $MAPbI_3$	1.09 (1.06±0.03)	22.4 (21.5±0.89)	78.1 (79.2±1.4)	19.08 (17.98±0.63)
$MAPbI_3$-TMTA before cross-link	1.11 (1.10±0.01)	22.8 (22.1±0.39)	80.2 (80.0±0.8)	20.22 (19.52±0.37)
$MAPbI_3$-TMTA after cross-link	1.09 (1.08±0.02)	22.7 (22.2±0.52)	78.2 (77.0±1.4)	19.26 (18.43±0.45)
The values in brackets are the average device parameters among 20 separated devices.				

**Supplementary Table 2│The ions and electronic conductivity of perovskite films measured through galvanostatic characterization.**			
**Conductivity (10^{-9} S cm^{-1})**	**Control $MAPbI_3$**	**$MAPbI_3$-TMTA before cross-link**	**$MAPbI_3$-TMTA after cross-link**
σ_{ion}	0.909	0.893	0.608
σ_{eon}	0.159	0.167	0.160
σ_{ion}: ions conductivity; σ_{eon}: electronic conductivity.			

Supplementary References

1. Domanski, K., Alharbi, E. A., Hagfeldt, A., Grätzel, M. & Tress, W. Systematic investigation of the impact of operation conditions on the degradation behaviour of perovskite solar cells. *Nat. Energy* **3**, 61-67, (2018).
